# Features of VEGF and IL-6 expression in patients with inflammatory breast cancer considering molecular-biological characteristics

**DOI:** 10.25122/jml-2022-0172

**Published:** 2023-01

**Authors:** Ivan Ivanovich Smolanka, Irina Yuriivna Bagmut, Oleksii Volodimirovich Movchan, Michael Ivanovich Sheremet, Oleksandr Mykolayovych Bilyi, Andriy Oleksandrovich Lyashenko, Irina Viktorivna Dosenko, Anton Dmitrovich Loboda, Oksana Mykolaivna Ivankova, Igor Leonidovich Kolisnyk

**Affiliations:** 1 National Cancer Institute, Ministry of Health, Kyiv, Ukraine; 2 Kharkiv Medical Academy of Postgraduate Education, Kharkiv, Ukraine; 3 Department of Surgery No.1, Bukovinian State Medical University, Chernivtsi, Ukraine; 4 VN Karazin Kharkiv National University, Ministry of Education, Kharkiv, Ukraine

**Keywords:** inflammatory breast cancer, vascular endothelial growth factor, interleukin-6, neoadjuvant chemotherapy personalization

## Abstract

Expression of pro-malignant factors (VEGF) and cytokines like inflammatory components support breast cancer development. We examined 46 patients with stage IIIB inflammatory breast cancer (IBC) and 24 with stage IIA-IIIB breast cancer (BC) without secondary edema. Hormone receptors, Her-2/neu, Ki-67 index, VEGF, and IL-6, were determined for all patients before and after neoadjuvant treatment. They associated the expression of VEGF for IBC patients with an unfavorable prognosis. VEGF level for IBC lymph node metastases was higher than in patients without such lesions (1.4 times), and there was a significant increase in VEGF levels in the G3 category of malignancy (1.54-fold increase). In IBC patients with positive HER2/neu status, VEGF levels were 1.51 times higher compared to those with negative HER2/neu status (r=0.36, p<0.05). IL-6 level during therapy in IBC patients remained high, which occurs in active tumor development. Comparative analysis of the VEGF/IL-6 ratio during treatment of patients with IBC was higher *vs*. IIIB stage breast cancer without edema (1.4 *vs*. 0.7), indicating the aggressiveness of the tumor process and confirmed by an objective response to treatment (regression<30%).

## INTRODUCTION

Breast cancer is a common type of cancer among women and a leading cause of cancer-related deaths globally [1]. By the end of 2020, 7.8 million women had been diagnosed with breast cancer in the previous five years, making it the most common disease in the world (World Health Organization) [2].

The edematous form of breast cancer is a type of inflammatory breast cancer (IBC) characterized by edema and/or redness of the breast skin. It accounts for 25% of IBC cases. According to the TNM classification of malignant tumors (8^th^ edition), IBC is designated T4d, and the secondary-edematous form is T4b [3]. Numerous studies have shown that the inflammatory components seen in the tumor microenvironment actively encourage locally advanced genesis and evolution of breast cancer [4]. This is particularly evident in patients with cutaneous edema and peritumoral tissues (T4b cases) and those with inflammatory breast cancer (T4d cases) [5].

Infiltration of monocytes into breast cancer is triggered by the production of monocyte chemoattractants CCL5 and CCL2. Tumor-associated macrophages may express pro-malignant mediators such as tumor necrosis factor-alpha, which may enhance the production of tumor-supporting molecules by tumor cells, such as matrix metalloproteinases [6]. This increased monocyte recruitment and activation of tumor-associated macrophages at the tumor site may result in increased levels of expression of pro-malignant factors such as angiogenic mediator vascular endothelial growth factor (VEGF) and cytokines. Chemokines may directly trigger some of these actions [7].

Inflammatory mammary breast carcinoma is a complex and aggressive histological pattern that poses significant surgical and oncological challenges. It tends to present as a large, palpable lump that is more advanced than the most common type of breast cancer, invasive ductal carcinoma [8]. By concentrating on a case report of IBC, our study illustrates the significance of being aware of histological grade. Breast surgeons should prioritize careful axillary examination due to the high likelihood of lymph node involvement in these cases, reflecting the lymphotropic behavior of this subtype [9].

Vascular endothelial growth factor (VEGF), known as the "angiogenic switch", is one of the most effective direct angiogenic factors [10]. VEGF enhances vascular permeability, one of the additional mechanisms of neo-angiogenesis, leading to the accumulation of blood plasma fibrin in tissues. The survival rate of activated endothelial cells and newly formed microvessels is also VEGF-dependent. In addition to promoting neo-angiogenesis, VEGF plays a role in maintaining the survival of new capillaries in tumors [11].

The purpose of this study was to use dynamic detection through neoadjuvant chemotherapy to assess the expression of the proangiogenic factor VEGF and the cytokine IL-6 in patients with inflammatory breast cancer, considering the main characteristics of the tumor in order to develop personalized therapy approaches.

## MATERIAL AND METHODS

We examined and treated 46 patients with stage IIIB inflammatory breast cancer (IBC) and 24 patients with stage IIA-IIIB non-edematous breast cancer from January 2017 to March 2021. The IBC patients were categorized into Luminal A (18 patients), Luminal B (10 patients), HER2/neu positive (11 patients), and triple-negative (7 patients), while non-edematous breast cancer patients were divided into Luminal A (10 patients), Luminal B (8 patients), HER2/neu positive (3 patients), and triple-negative (3 patients). The ages of the patients ranged from 38 to 73 years, with a median age of 58 years.

The hormonal receptors, HER2/neu status, Ki-67 index in tumor tissue, VEGF, and IL-6 expression in blood serum was determined for all patients. Standard neoadjuvant treatment was performed following the National Comprehensive Cancer Network (NCCN) protocols. After therapy, VEGF and IL-6 levels were determined again. The following antibodies were used for immunohistochemical determination of estrogen, progesterone receptors, HER2/neu, and Ki-67 index. ABC-Kit (universal) from Novocastra was used as the visualization system. Pharmab DAB was used to detect the coloring of the reaction result. The level of VEGF and IL-6 was determined by enzyme-linked immunosorbent assay (ELISA) using standard sets of reagents. Before and after neoadjuvant therapy, blood samples were collected to measure the levels of VEGF and IL-6. Tumor regression was evaluated using the Response Evaluation Criteria in Solid Tumors (RECIST).

### Statistical analyses

The data were analyzed using the Statistical Package for the Social Sciences (SPSS) 22.0 software. Pearson correlation was used to examine the relationship between IBC markers (edema) and the outcome variable (VEGF/IL-6 ratio) in the specified direction. The Cohen coefficients of 0.10, 0.30, and 0.50 indicate small, medium, and large effect sizes, respectively. The VEGF/IL-6 ratio was considered the dependent variable, and the relationship between edema and the VEGF/IL-6 ratio was controlled for potential confounding factors such as histological type, receptor status, tumor localization, lymph node lesions, degree of malignancy, Ki-67 index, HER2/neu status, and menopause status. The results were reported using a 95% bias-corrected confidence interval. A zero value outside the interval indicated a statistically significant indirect effect (p<0.05). The mediation analysis was performed using the Hayes Process.

## RESULTS

The results suggest that VEGF levels were correlated with ER negativity, higher lymph node involvement, and HER2/neu positivity. On the other hand, IL-6 levels appeared to be primarily impacted by menopausal status. The median VEGF level in the blood of 46 IBC patients before treatment was 441 pg/ml, with a range of 267 to 541 pg/ml. The median IL-6 level was 9.5 pg/ml, ranging from 1.87 to 18.7 pg/ml. The data before treatment, based on the main clinical and morphological characteristics of IBC, is presented in Table 1.

**Table 1:** VEGF content in the serum of IIIB stage IBC patients depending on the clinical and morphological characteristics of the disease.

Group	VEGF (normal range 0–115), pg/ml		IL-6, (normal range <1.5), pg/ml
	n	Median	Median
**Patients with IBC**	46	441	9.4
**Menopause status**			
Preserved menstrual function	10	358	3.8
Premenopause	11	382	5.9
Postmenopause	25	427	10.2 ^#^
**Histological type**			
Invasive ductal cancer	35	399	8.2
Invasive lobular cancer	11	412	6.9
**Localization**			
Left breast	21	431	9.1
Right breast	25	412	8.4
**The size of the tumor**			
<5 cm	26	397	7.4
>5 cm	20	421	9.8
**Lymph node lesions (criterion N)**			
N-	18	346	7.1
N+	28	489 ^#^	8.2
**The degree of malignancy**			
G2	7	321	6.1
G3	39	496	8.1
**Ki-67 index**			
<25%	14	312	6.4
>25%	32	492 ^#^	7.8
**ER status**			
Positive	26	329	7.9
Negative	20	496	8.8
**PR status**			
Positive	18	338	6.9
Negative	28	491 ^#^	8.1
**Her-2/neu status**			
Negative	35	328	8.6
Positive	11	498 ^#^	10.2

# – the significance of differences in groups regarding distribution by clinical and morphological parameters, (Wilcoxon criterion), p<0.05.

No significant correlation was found between VEGF content, menopausal status, localization, and tumor size in the serum of patients with IBC between 37–76 years. IL-6 concentration in the serum in postmenopausal women was higher compared to pre-menopausal patients.

Most patients (76%) had invasive ductal cancer, with a median VEGF level of 399 pg/ml and a median IL-6 level of 8.1 pg/ml. The remaining 24% of patients had invasive lobular cancer, with a median VEGF content of 412 pg/ml and a median IL-6 level of 6.9 pg/ml. The concentration of IL-6 was slightly higher in patients with ductal infiltrative breast cancer compared to those with lobular infiltrative cancer.

The correlation analysis between VEGF expression and lymph node damage (criterion N) revealed that the VEGF level was 1.4 times higher in IBC patients with lymph node metastases compared to those without such lesions. A similar pattern was observed for IL-6.

The correlation between VEGF levels and tumor-grade malignancy was analyzed. 85% of patients had a low-grade malignancy (G3) and had 1.54 times higher VEGF levels compared to the 15% of patients with a moderate grade (G2) (r=0.48, p<0.05). The increase in IL-6 was slightly higher (1.3-fold) in patients with a low grade of malignancy (G3).

As we can see from Table 1, the level of VEGF correlated with the expression of the index Ki- 67, which characterizes the proliferative activity of the tumor. Thus, in 70% of patients, this indicator was >25%, while in 30% of patients, it was <25%. Patients with a Ki-67 index expression level higher than 25% in the tumor showed a 1.6-times increase in serum VEGF content compared to those with a level less than 25% (r=0.49, p<0.05). However, IL-6 levels showed no significant differences. It is known that a lower content of the Ki-67 index (<25%) indicates a less aggressive tumor. A higher Ki-67 index, which reflects a higher level of tissue nuclear antigen of proliferating cells, generally responds better to hormone therapy in the presence of positive PR and ER. Thus, an analysis of the relationship between VEGF and Ki-67 indexes levels revealed an increased VEGF content in the Ki-67 index overexpression group, which may show the aggressiveness of the disease.

The analysis of the hormone receptor expression in the tumor showed that 57% of patients with IBC had a positive status of ER, while 43% had a negative ER status. Similarly, 39% of patients had a positive PR status, while 61% had a negative PR status. The study of the relationship between VEGF levels and hormone receptor status showed that VEGF levels were higher in ER-negative tumors (1.5 times more) than in ER-positive tumors (r=0.46, p<0.05). Similarly, VEGF levels were higher in PR-negative tumors (1.45 times more) than in PR-positive tumors (r=0.37, p<0.05). However, no correlation was found between IL-6 levels and the presence of ER and PR receptors.

The results of the HER2/neu expression immunohistochemistry test showed that 24% of tumors were HER2/neu positive (3+) and 76% were HER2/neu negative (0-2+). Overexpression of HER2/neu in some types of breast cancer leads to increased proliferation, angiogenesis, and impaired apoptosis regulation. This overexpression is linked to more aggressive disease, higher metastatic potential, and a poor prognosis. The relationship between VEGF levels and HER2/neu tumor expression was studied in IBC patients, revealing that VEGF content was 1.51 times higher in the HER2/neu positive tumor group compared to the HER2/neu negative group (r=0.36, p<0.05). This indicates that it may be possible to predict the course of the tumor in IBC patients.

**Figure 1:**
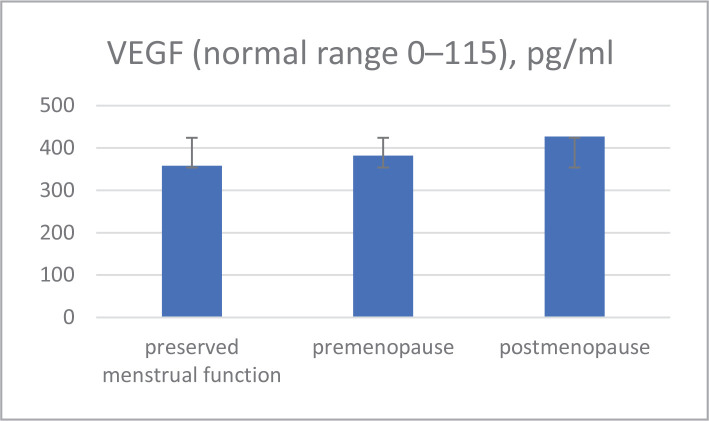
Menopause status and VEGR correlation.

Therefore, expression of VEGF in patients with IBC was associated with factors of unfavorable prognosis (degree of tumor malignancy, proliferative activity of the tumor (Ki-67 index), and hormone resistance (absence of steroid hormone receptors) (Figure 1).

**Figure 2:**
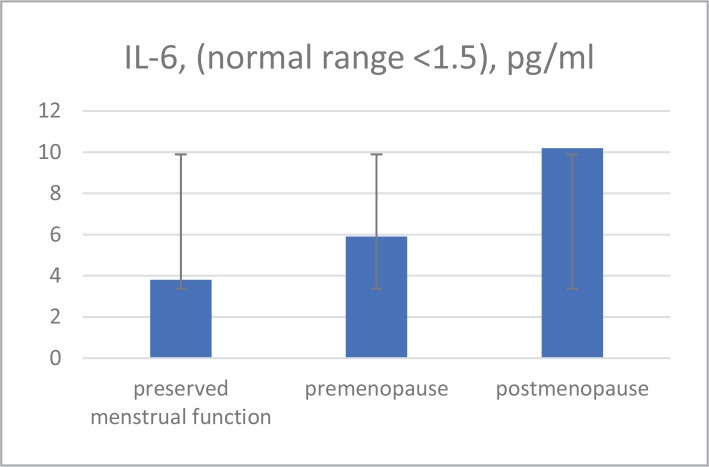
Menopause status and IL-6 correlation.

The study of IL-6 levels in the serum of patients with IBC, considering clinical and morphological factors, revealed a significant association with post-menopause status (Figure 2).

IBC were stratified into two subgroups based on the answer of neoadjuvant therapy: (1) insufficient effectiveness (progression of the disease) and (2) stabilization or partial regression of the cancer process.

**Figure 3:**
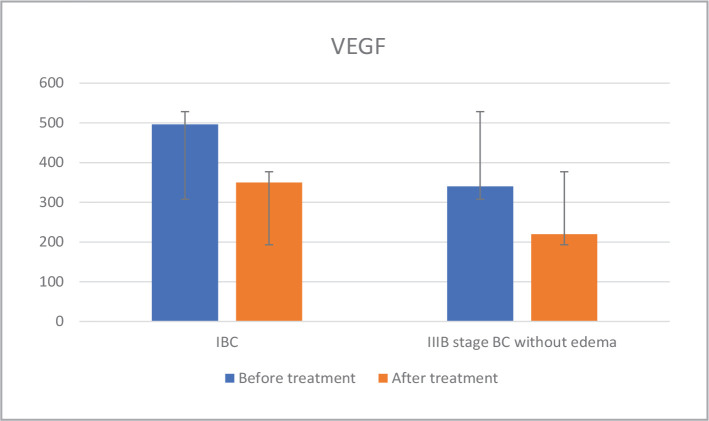
VEGF dynamics before and after chemotherapy.

The level of proangiogenic factor VEGF was analyzed during treatment, and it was found that in 60% of IBC patients (subgroup 1), the VEGF level remained high (350 pg/ml) after therapy, indicating the tumor's aggressiveness and ineffective neoadjuvant treatment. In contrast, the VEGF level decreased 1.2 times in the other 40% of patients (subgroup 2) (Figure 3). In patients with IIIB stage BC without edema (subgroup 3), the VEGF level decreased by 1.54 times after treatment.

**Figure 4:**
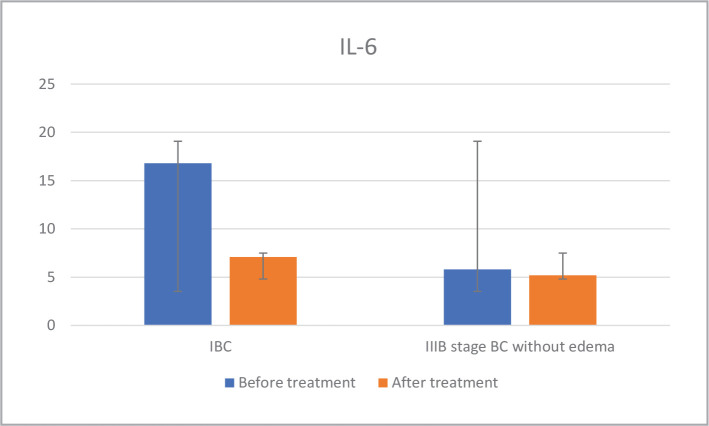
IL-6 dynamics before and after chemotherapy.

The analysis of IL-6 levels in the dynamics of neoadjuvant therapy in IBC patients revealed that the first subgroup still had elevated levels, typically seen during active tumor growth. The cytokine levels in the second subgroup decreased close to normal levels after treatment. In the third subgroup of IIIB stage BC patients without edema, the IL-6 levels remained within the normal range both before and after therapy - as depicted in Figure 4.

Then, we performed a correlation between VEGF and IL-6 for each subgroup. The connection analysis between the levels of the investigated factors, the VEGF/ IL-6 ratio before and after treatment separately for each subgroup was calculated as K1 before treatment and K2 after treatment (Table 2). Results showed that in the first subgroup, a K2/K1 ratio greater than 1 was associated with a more aggressive tumor process, as evidenced by a less than 30% regression in response to treatment. Table 3 displays the correlation between the K2/K1 ratio and regression.

**Table 2:** The content of IL-6 and VEGF in the serum of IBC patients and IIIB stage BC without edema in the dynamics of neoadjuvant therapy.

Patients	n (subgroup)	VEGF (normal range 0-115), pg/ml		IL-6, (normal range <1.5), pg/ml		K2/K1
		Before treatment	After treatment	Before treatment	After treatment	
**IBC**	24 ^#^	496	350	16.8	7.1	1.4
**IIIB stage BC without edema**	22	340	220	5.8	5.2	0.7

# – the significance of differences in groups regarding distribution by clinical effects and the RECIST data (Wilcoxon criterion), p<0.05.

**Table 3:** Correlation between VEGF/IL-6 ratio coefficients and the degree of regression of the tumor in IBC patients and IIIB stage BC without edema in the dynamics of neoadjuvant therapy.

Groups of patients (K2/K1)	Regression >30%, abs., (%)	Regression <30%, abs., (%)
**IBC (K2/K1=1.4)**	3 (11%)	25 (89%)
**IBC (K2/K1=1.04)**	7 (40%)	11 (60%)
**BC without edema (K2/K1=0.7)**	19 (80%)	5 (20%)

## DISCUSSION

According to previous research, there is a parallel increase in VEGF and IL-6 levels observed in patients with cancer, similar to the results seen in our study. The expression of mRNA is regulated by IL-6, as reported by Subotički T [12], with an increased amount of immune-related signaling in response to VEGF.

The process of VEGF is carried out through the phosphoinositide-3-kinase pathway. The mechanism was confirmed by using a recombinant humanized monoclonal antibody directed against the human interleukin-6 receptor (IL-6), which impedes the action of IL-6. As demonstrated by Briukhovetska D [13], IL-6 is capable of inducing angiogenesis through the activation of NF-κB, leading to the secretion of VEGF by cancer cells, promoting cell proliferation, invasion, and inhibiting apoptosis.

In the second subgroup of patients, the ratio of K2/K1 (1.04) showed lower levels of IL-6 before and after treatment, indicating a milder inflammatory process. The central role of IL-6 in promoting and supporting inflammation highlights its negative impact on the tumor. In the third subgroup of patients with IIIB stage BC without edema, the ratio K2/K1 (0.7) suggests a less pronounced inflammatory process. This is reflected in the positive outcomes of the non-adjuvant treatment, as reported by Baram T [14], who found that pro-inflammatory components enhance cancer cell plasticity by increasing stemness/EMT, treatment resistance, and dormancy exit.

Therefore, our study confirmed the correlation between elevated VEGF levels and increased synthesis of IL-6 in patients with inflammatory breast cancer (IBC), aligning with the findings of Tawara K [15] which showed the importance of VEGF and IL-6 co-expression in poor survival of IBC patients. The inflammatory processes in the breast are viewed as a favorable environment for tumor cell metastasis, and VEGF expression facilitated by IL-6 exacerbates this. The combined effect of IL-6 on tumor growth can serve as a basis for targeted therapeutic interventions. The whole complex influence of IL-6 on tumor growth can become the basis of the purposefulness of carrying out various therapeutic actions. The enhancement of IL-6 synthesis occurs against the background of other pro-inflammatory cytokines and is a mediator of VEGF angiogenesis. Therefore, new methodological approaches are being laid, which are based on the complex use of drugs having antitumor and anti-inflammatory activities.

Thus, it has been shown that in most patients with IBC, there is a parallel increase in the levels of the proangiogenic factor VEGF and the pro-inflammatory cytokine IL-6. The relationship between these factors shows the aggressiveness of the tumor process accompanied by inflammation as an unfavorable background for malignancy progression and negative prognosis. This explains the interest in finding therapeutic approaches that aim to influence IL-6 and VEGF to reduce inflammation and slow angiogenesis, which will increase the effectiveness of neoadjuvant treatment. The K2/K1 ratio from our study may predict the aggressiveness of the IBC.

## CONCLUSION

Most patients with IBC before neoadjuvant therapy had high levels of the proangiogenic factor VEGF (median 441 pg/ml) and the pro-inflammatory cytokine IL-6 (median 9.4 pg/ml), indicating the aggressive nature of the tumor growth and its association with inflammation. The relationship between the concentration of the proangiogenic factor VEGF, pro-inflammatory cytokine IL-6, their correlation, and the objective response to treatment (by the degree of regression), which evaluated the direct effect of neoadjuvant therapy in patients with IBC, was determined. Comparative analysis of changes in the ratio of VEGF/IL-6 during neoadjuvant treatment of patients with IBC and IIIB stage BC without edema showed that after treatment, the ratio was higher in most patients with IBC (1.4 *vs*. 0.7 in BC), indicating aggressiveness of the tumor process and confirmed by an objective response to treatment (regression <30%).

## Personal thanks

We want to thank Professor Irina Yurievna Bagmut.
